# Estimated Intakes and Sources of Total and Added Sugars in the Canadian Diet

**DOI:** 10.3390/nu6051899

**Published:** 2014-05-08

**Authors:** Tristin D. Brisbois, Sandra L. Marsden, G. Harvey Anderson, John L. Sievenpiper

**Affiliations:** 1 Nutrition Information Service, Canadian Sugar Institute, 10 Bay Street, Ste. 620, Toronto, ON M5J 2R8, Canada; E-Mail: smarsden@sugar.ca; 2 Nutritional Sciences and Physiology Director, Program in Food Safety, Nutrition and Regulatory Affairs, Department of Nutritional Sciences, Faculty of Medicine University of Toronto, Toronto, ON M5S3E2, Canada; E-Mail: harvey.anderson@utoronto.ca; 3 Resident Physician (PGY-4), Department of Pathology and Molecular Medicine, Faculty of Health Sciences, McMaster University, HSC-2N22B, 1200 Main St. W, Hamilton, ON L8N 3Z5, Canada; E-Mail: john.sievenpiper@medportal.ca; 4 Knowledge Synthesis Lead Toronto 3D Knowledge Synthesis and Clinical Trials Unit, Clinical Nutrition and Risk Factor Modification Centre, St. Michael’s Hospital, University of Toronto, #6130-61 Queen Street East, Toronto, ON M5C 2T2, Canada

**Keywords:** added sugars, sucrose, high fructose corn syrup, consumption, availability, trends, Canada, food intake

## Abstract

National food supply data and dietary surveys are essential to estimate nutrient intakes and monitor trends, yet there are few published studies estimating added sugars consumption. The purpose of this report was to estimate and trend added sugars intakes and their contribution to total energy intake among Canadians by, first, using Canadian Community Health Survey (CCHS) nutrition survey data of intakes of sugars in foods and beverages, and second, using Statistics Canada availability data and adjusting these for wastage to estimate intakes. Added sugars intakes were estimated from CCHS data by categorizing the sugars content of food groups as either added or naturally occurring. Added sugars accounted for approximately half of total sugars consumed. Annual availability data were obtained from Statistics Canada CANSIM database. Estimates for added sugars were obtained by summing the availability of “sugars and syrups” with availability of “soft drinks” (proxy for high fructose corn syrup) and adjusting for waste. Analysis of both survey and availability data suggests that added sugars average 11%–13% of total energy intake. Availability data indicate that added sugars intakes have been stable or modestly declining as a percent of total energy over the past three decades. Although these are best estimates based on available data, this analysis may encourage the development of better databases to help inform public policy recommendations.

## 1. Introduction

With global concerns regarding obesity and excess energy availability, trends in added sugars consumption have been suggested to be linked to obesity and associated chronic diseases [1]. Understanding national consumption levels and recent trends is essential for program and policy considerations. Both food supply data and dietary surveys are important in providing country-specific data to monitor estimated added sugars intakes in relation to trends in caloric intake and obesity. Unfortunately, there are relatively few published studies reporting added sugars intakes. This may be due in part to the difficulties in estimating added sugars consumption. National food supply (availability) economic data can be used to estimate trends in added sugars consumption, however these data represent the amount purchased not consumed, and do not account for losses. To correct for this, some reports adjust availability data for waste (e.g., retail, institutional and household losses) to provide an estimate of apparent consumption, but this approach is recognized to still underestimate wastage [2].

While availability data are useful to indicate trends, they provide little insight into individual consumption patterns or variability of intake within the population. For this, food intake data from dietary surveys needs to be combined with accurate data on the sugars content in foods. The lack of a comprehensive database of added sugars content in foods provides an additional complication, thus limiting analyses to total sugars. Further, the global variation in terminology used to describe sugars also makes it difficult to compare estimated intakes across countries. In Canada, by definition, the term “sugar” describes sucrose from sugar cane or beets [3]. “Added sugars” describes sugars (or ingredients that functionally substitute for sugars) that are added to foods, while “sugars” or “total sugars” describes all sugars, both naturally occurring and added [4].

A recent Canadian Health Report [5] has described total sugars consumption based on Canadian Community Health Survey (CCHS) national food intake survey data. Therefore the purpose of this article was to estimate and trend added sugars intakes and their contribution to total energy intake among Canadians by first, using CCHS nutrition survey data of intakes of sugars in foods and beverages and second, using Statistics Canada availability data and adjusting for wastage to estimate intakes. Sources of sugars and their contribution to total energy among children, adolescents and adults are reported.

## 2. Experimental Section

### 2.1. Data Sources

#### 2.1.1. Survey Data

In 2004, the CCHS collected food and nutrient intakes of 35,107 Canadians using a 24 h dietary recall. Included in the analysis were 34,386 respondents; excluded were children <1 year, respondents with invalid dietary recalls, pregnant or breastfeeding women, and children being breastfed. This was the only national dietary survey conducted in Canada in the last 30 years. Intakes were based on all foods and beverages reported, the composition of which was calculated using Health Canada’s Canadian Nutrient File [6]. A detailed description of the survey and methods are described elsewhere [5,7]. For this article, added sugars intakes were estimated from CCHS data of total sugars and energy intakes, previously published by Health Canada and Statistics Canada [5,7]. The mean values were used in these analyses.

#### 2.1.2. Availability Data

Annual availability data (Also known as disappearance data, this reflects the total amount of a food or commodity entering the market, regardless of its final use) of “*sugars and syrups*” (kilograms per person), soft drinks (litres per person; kilocalories per person), and total food energy (kilocalories per person) in Canada were obtained from the Statistics Canada CANSIM database (Tables 002-0011 (years 1966–2012) and 003-0080 (years 1976–2009)) [8]. CANSIM is a multidimensional database which tracks various socioeconomic statistics of Canadians. The “*sugars and syrups*” category includes refined sugar (Includes all white, brown and specialty sugars and sugar syrups made from sugar cane or sugar beets), honey and maple sugars, but does not include corn sweeteners (e.g., high fructose corn syrup, HFCS). The soft drink category included sweetened carbonated beverages, but not sports drinks or other sweetened drinks. For comparison purposes, annual availability data of regular (caloric) soft drinks (gallons per person) in the US were obtained from the US Department of Agriculture (USDA) Economic Research Service Food Availability (Per Capita) Data System (Food Availability, Beverages) [9]. From these data, apparent added sugars consumption was calculated by adjusting availability data for sizable losses that occur in distribution, storage, preparation and consumption (e.g., discarded or spoiled) or by presenting availability data as a percent of total energy. The linear trend in apparent sugar consumption since 1966 was evaluated by ANOVA.

### 2.2. Data Analyses

#### 2.2.1. Survey Data

Statistics Canada has published CCHS data (from 24 h food recall) on total sugars intake and the top sources of sugars in Canadian children (1–8 years); adolescents (9–18 years); and adults’ (19+ years) diets [5]. The top sources accounted for 84%–86% of total sugars intake. For the present article, estimates of added sugars intake were derived from these top sources by categorizing them based on the majority of sugars being either naturally occurring or added. It was assumed that all sugars in fruits, vegetables, milk, and 100% fruit juice were naturally occurring, while all sugars in confectionary, sugars, fruit drinks and cereals/grains were considered added. A high percentage of the sugars in fruit drinks may be naturally occurring; however the exact range is unknown and therefore all sugars in fruit drinks were considered to be added for a conservative estimate. CCHS data defined “milk” as whole, 2%, 1%, skim, evaporated, condensed, and other (soya, goat, whey, buttermilk), but did not include flavoured milk or other milk products (e.g., yogurt). Therefore no added sugars were present in the milk category. The sugars from the top sources were summed for each of the naturally occurring and added sugars categories. From these data, the proportion of added sugars from total sugars was estimated for each age group (children 1–8 years, adolescents 9–18 years, adults 19+ years); it was assumed that the proportion of added sugars in the top sources would remain consistent for the remaining 14%–16% sources of sugars in the diet. Added sugars were expressed as a percentage of total energy for each age group and all averages were weighted based on population sample numbers [7].

CCHS data for intakes (average and range) of carbohydrate, total sugars, and added sugars were compared to the dietary recommendations described in the Dietary Reference Intakes (DRI) report [10]. Averages were weighted and based on intakes among Canadians of all ages. Ranges represented the range in weighted averages among the three age groups (children 1–8 years, adolescents 9–18 years and adults 19+ years).

#### 2.2.2. Availability Data

Apparent consumption (*i.e.*, availability data adjusted for losses) was calculated using a 40% waste adjustment factor based on current estimates of food waste (40%) [2] and the USDA estimated loss factor for added sugars and sweeteners (41%, updated through 2010) [11]. Statistics Canada continues to apply a waste adjustment factor of approximately 30% to estimate consumption [8]; however, this adjustment is based on USDA’s data and studies from the mid-1970s and earlier and does not reflect USDA’s more recent estimate based on updated loss estimates at the primary, retail and consumer levels.

Statistics Canada annual availability data [8] on “*sugars and syrups*”, soft drinks, and total energy (kcal) were used to examine consumption trends of added sugars over time. Availability data for corn sweeteners is not reported to Statistics Canada as this is proprietary information. Because the main use of HFCS is to sweeten caloric beverages, soft drink data was used as an indirect estimate of HFCS availability and trends. “*sugars and syrups*” and soft drink (HFCS) availability data (*i.e.*, caloric contribution from the sugars) were combined to estimate added sugars consumption in Canada. Soft drink availability data assumes all soft drinks are caloric (*i.e.*, doesn’t account for the proportion of diet drinks), which overestimates the amount of HFCS consumed. The extent to which this overestimation serves to balance the unreported amount of HFCS in other foods and beverages (e.g., fruit drinks) not captured in the analysis is unknown.

Statistics Canada discontinued publication of energy available from the food supply in 2009 (commenced in 1976). The reasons for this have not been published but are assumed to be fiscal. Trends are reported up to this date. To account for annual variability, an average of the last five available years of Statistics Canada data for “*sugars and syrups*”, soft drinks, and total energy were used to estimate added sugars consumption. USDA availability data for soft drinks were discontinued after 2007 [9]; trends are reported up to this date. Data were converted from gallons to litres.

## 3. Results

### 3.1. Survey Data

In 2004, the CCHS reported total sugars (naturally occurring and added) to contribute an average 116 g/day or approximately 21% of total energy intake among Canadians of all ages. The top 10 foods, accounting for 85% of total sugars intake, are shown in Table 1. Children (ages 1–8 years) received a higher percentage of sugars from natural sources, whereas adolescents (9–18 years) received a higher percentage from added sugars. For adults, CCHS data indicate that approximately half of the energy from total sugars came from added sugars. For children and adolescents, added sugars accounted for 39% and 57% of total sugars, respectively. Using these proportions, estimated added sugars intake among all Canadians contributed an average 11% of total energy, ranging from 10% to 14% (Table 1).

**Table 1:** Top sources of total sugars intake by age group, categorized by estimated naturally occurring or added sugars according to Canadian Community Health Survey (2004) data.

Children aged 1–8 years				
Category *	CCHS % total sugars	CCHS Total Sugars % Energy	Est Naturally Occurring Sugars % Energy	Est Added Sugars % Energy
Milk	19.9	5.3	5.3	
Fruit	14.9	4.0	4.0	
Fruit juice	14.6	3.9	3.9	
Confectionary	8.7	2.3		2.3
Fruit drinks	6.2	1.6		1.6
Sugars (white and brown)	5.4	1.4		1.4
Other sugars (syrups, molasses, honey, *etc.*)	5.3	1.4		1.4
Cereals, grains and pasta	4.3	1.2		1.2
Soft drinks—regular	3.6	1.0		1.0
Vegetables	2.9	0.8	0.8	
**TOTAL Top 10**	**85.8**	**22.8**	**13.9**	**8.9**
Other food categories	14.2	3.8	2.3	1.5
**Total Foods**	**100.0**	**26.6**	**16.2**	**10.4**
**Adolescents aged 9–18 years**				
Category *	CCHS % total sugars	CCHS Total Sugars % Energy	Est Naturally Occurring Sugars % Energy	Est Added Sugars % Energy
Soft drinks—regular	14.3	3.5		3.5
Milk	14.0	3.5	3.5	
Fruit	10.6	2.6	2.6	
Confectionary	10.3	2.6		2.6
Fruit juice	9.1	2.3	2.3	
Fruit drinks	7.4	1.8		1.8
Sugars (white and brown)	6.3	1.6		1.6
Other sugars (syrups, molasses, honey, *etc.*)	5.4	1.4		1.4
Cereals, grains and pasta	4.5	1.1		1.1
Vegetables	3.3	0.8	0.8	
**TOTAL Top 10**	**85.3**	**21.2**	**9.2**	**12.0**
Other food categories	14.7	3.7	1.6	2.1
**Total Foods**	**100.0**	**24.9**	**10.8**	**14.1**
**Adults aged 19+ years**				
Category *	CCHS % total sugars	CCHS Total Sugars % Energy	Est Naturally Occurring Sugars % Energy	Est Added Sugars % Energy
Fruit	17.4	3.5	3.5	
Soft drinks—regular	13.0	2.6		2.6
Sugars (white and brown)	11.4	2.3		2.3
Milk	10.7	2.1	2.1	
Fruit juice	7.6	1.5	1.5	
Vegetables	6.8	1.4	1.4	
Confectionary	5.3	1.1		1.1
Other sugars (syrups, molasses, honey, *etc.*)	4.5	0.9		0.9
Fruit drinks	3.7	0.7		0.7
Cereals, grains and pasta	3.3	0.7		0.7
**TOTAL Top 10**	**83.8**	**16.7**	**8.5**	**8.3**
Other food categories	16.2	3.3	1.6	1.6
**Total Foods**	**100.0**	**20.0**	**10.1**	**9.9**

* Categorized based on the majority of sugars being either added or naturally occurring. Top ten sources represent 84%–86% of total sugars intake. Added sugars accounted for 39% of total sugars for children, 57% for adolescents and 50% for adults. Milk included all forms of milk reported: whole, 2%, 1%, skim, evaporated, condensed, and other types of milk (soya, goat, whey, buttermilk); Confectionary included candy, gum, popsicles, sherbert, jello, dessert toppings, pudding mixes, and chocolate bars**;** Fruit included citrus fruits (oranges, grapefruits, *etc.*), apples, bananas, cherries, grapes and raisins, melons (cantaloupe, honeydew, watermelon), peaches, nectarines, pears, pineapple, plums and prunes, strawberries, and other fruits (blueberries, dates, kiwis, fruit salads, dry fruit, *etc.*); Cereals, grains and pasta included pasta, rice, cereal grains and flours, whole grain, oats, and high-fibre bread, and breakfast cereals (other); Vegetables included beans, broccoli, cabbage and kale, cauliflower, carrots, celery, corn, lettuce and leafy greens (spinach, mustard greens, *etc.*), mushrooms, onions, green onions, leeks, garlic, peas and snow peas, red and green peppers, squashes, tomatoes, tomato and vegetable juices, potatoes, and other vegetables (cucumber, immature beans, brussel sprouts, beets, turnips). Abbreviations: CCHS: Canadian Community Health Survey; est: estimate. Source: 2004 Canadian Community Health Survey—Nutrition [7], adapted from *Statistics Canada, Sugar consumption among Canadians of all ages* [5].

**Figure 1:**
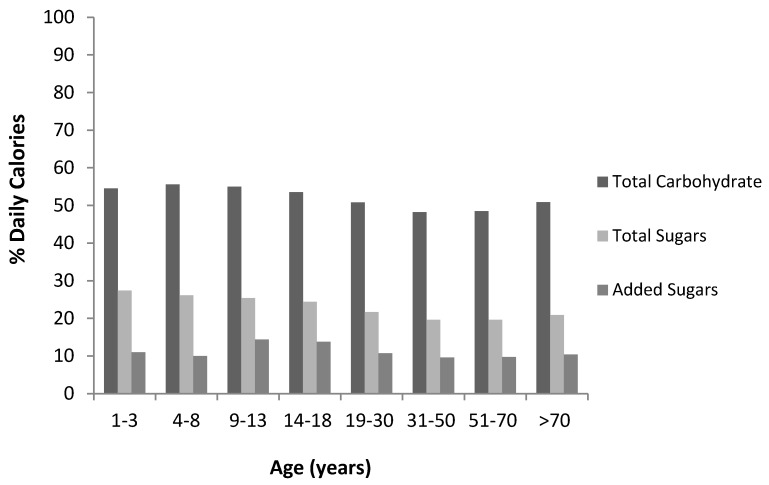
Average daily total carbohydrate, total sugars, and estimated added sugars intake (each as % calories) among Canadians according to Canadian Community Health Survey (2004) food intake data [5,7]. It should be noted that total carbohydrates consist of total sugars, and total sugars includes added sugars; therefore the bars are not to be summed.

Both total carbohydrate and total sugars intakes declined with age as a proportion of total calories consumed (Figure 1). Added sugars intake peaked during adolescent years, but otherwise remained relatively stable across the lifespan. Total sugars accounted for less than half (44%) of carbohydrate intake; added sugars accounted for less than a quarter. Average intakes of carbohydrate, total sugars and added sugars among Canadians were within dietary recommendations (Table 2).

**Table 2:** The average and range of carbohydrate, sugars, and added sugars intakes among Canadians according to Canadian Community Health Survey (2004) data compared to dietary recommendations.

	Average	Range	AMDR
**Carbohydrate**	51%	49%–56%	45%–65%
**Total sugars**	21%	19%–27%	None
**Added sugars (est)**	11%	10%–14%	<25%

AMDR based on Institute of Medicine Dietary Recommendation report (Canadian and US dietary guidelines) [10]. There is no dietary recommendation for total sugars or added sugars; the suggested maximum intake of added sugars is 25% of total energy. Abbreviations: AMDR = Acceptable Macronutrient Distribution Range; est: estimate.

### 3.2. Availability Data

**Figure 2:**
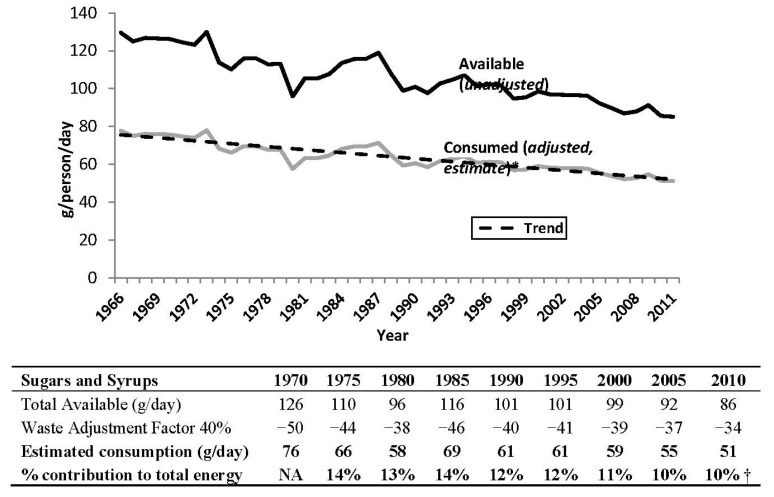
Sugars and syrups available for consumption (unadjusted availability data) and apparent consumption (adjusted availability data) in Canada from 1966 to 2011. Source: Statistics Canada (2012). * Experimental data, use with caution. Availability data have been adjusted for retail, household, cooking and plate loss using a 40% waste adjustment factor to calculate apparent consumption (consumed estimate) [2,11]. Data includes sugar, honey and maple sugars, and excludes corn sweeteners (*i.e.*, high fructose corn syrup (glucose-fructose), glucose syrup, and dextrose); high fructose corn syrup was introduced in the 1970s and has been the primary sweetener in soft drinks in Canada since the late 1990s. **†** Energy availability data was discontinued in 2009; value of 10% is an average of the last available five years (2005–2009).

**Figure 3:**
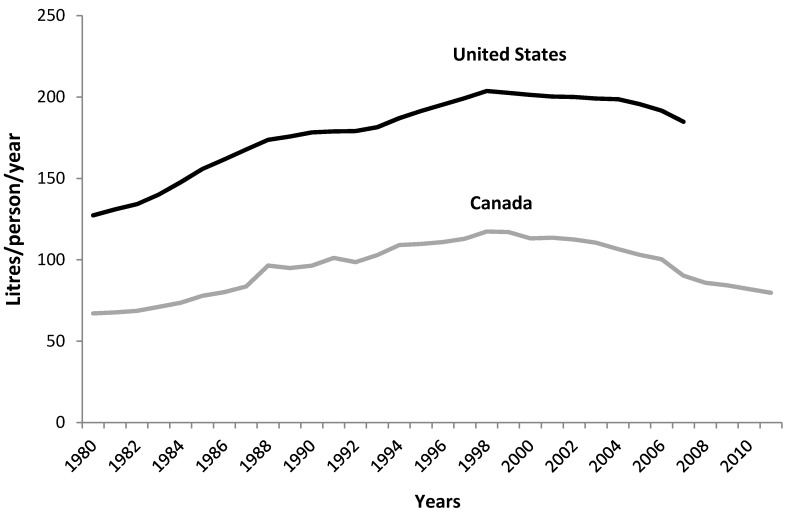
Soft drinks available for consumption (unadjusted) per capita from 1980 to 2011 in Canada and the United States. Canadian soft drink availability data includes both caloric and non-caloric soft drinks. For comparison, US data also includes both caloric and non-caloric soft drinks. Canadian diet drink share is not available; however, US share was 31% in 2007. Sources: Statistics Canada (2012) and United States Department of Agriculture (USDA) (2007). USDA soft drink data was discontinued in 2007.

Statistics Canada data show that the estimated apparent consumption (adjusted availability data) of “*sugars and syrups*” (“Sugars and syrups” category includes sugar from sugar cane and sugar beets, honey, and maple sugars, but does not include corn sweeteners (HFCS).) has been decreasing over the past four decades (*p* < 0.001, Figure 2). In 2012, apparent consumption of added “*sugars and syrups*” was 51 g/day, down from 76 g/day in 1970 (Figure 2). As a percentage of total energy, “*sugars and syrups*” declined from 14% in 1975 to 10% in 2010 (Figure 2). Soft drink availability in Canada increased from 1980 to 1998 but has declined over the past decade. The trend in the US is very similar; however soft drink consumption in the US is approximately double that in Canada (Figure 3). Canadian availability data show that the contribution of soft drinks (proxy for HFCS) to total energy availability was ~3%, down from the peak of 4% in the mid-1990s. By combining the caloric contribution from soft drinks (estimated HFCS) with that from “*sugars and syrups*”, added sugars can be calculated to contribute approximately 13% of total energy available in the food supply (Table 3). This estimate is similar, albeit slightly higher, than estimated added sugars intake from CCHS intake data (11%, Figure 1). These data suggest a stable or modest decline of total added sugars intake from 14% in the early 1970s (when HFCS was first introduced into the food supply) to 11%–13% in 2010 (Figure 2).

**Table 3:** Estimated average energy available from total added sugars in Canada for the last available five years (2005–2009).

	Energy (kcal)
Sugars and syrups	348
Soft drinks (HFCS)	109 *
Total added sugars	456
Total energy availability	3390
**% Energy total added sugars**	**13%**

* Overestimate as does not correct for diet soft drinks (*i.e.*, all soft drinks are considered caloric). Source: Statistics Canada (2012). Energy availability data was discontinued in 2009. Abbreviations: HFCS: high fructose corn syrup.

## 4. Discussion

This is the first report of estimated added sugars intakes among Canadians. Availability data suggest added sugars consumption in Canada to be stable or modestly declining as a percentage of total energy. Analysis of both survey and availability data suggests that added sugars average about 11%–13% of total energy intake. This is similar to consumption trends observed in Australia [12] and about three percentage points lower on average than US intakes of added sugars (2005–2010) for all age groups [13,14]. In absolute amounts, Canadian adults consume almost one third less added sugars compared to the US adult population (52 g *versus* 77 g/day, respectively) [14].

The overall decline in “*sugars and syrups*” availability, in part reflects the replacement of sugar by HFCS in sweetened beverages. The transition from sugar to HFCS, which started in the 1970s, was gradual and depended on the relative prices of the two sweetening agents. This caused annual variations in “*sugars and syrups*” availability, which can be seen until the late 1990s (Figure 2). Sugar has now been fully replaced by HFCS in almost all sweetened beverages in Canada, so there is much less annual variability. The previous variability in usage of HFCS and sugar in soft drinks did not allow for a precise trend in added sugars intake over the last 30 years. Still, with the decline of soft drink intake over the past decade and the overall decline of “*sugars and syrups*” availability, Statistics Canada data show that added sugars consumption has been stable or modestly declining as a percentage of total energy. Further, as food waste has progressively increased over the past four decades [2], early estimates for “*sugars and syrups*” availability may be higher than those reported here given our use of a 40% waste factor across all years.

Taken together, availability data indicate a modest decline of added sugars consumption in Canada. Similar trends have been observed in other developed countries, including Australia and the UK where added sugars intakes have fallen over the last 25 years [15,16,17]. In the US intakes of sugars declined 23% between 1999 and 2008 [18]. However, values for sugar supply on the global market are often quoted [1], which do not reflect country specific trends nor dietary intakes [19]. References to increasing global availability of added sugars can also be misleading when the vast majority of this growth reflects global population growth and development. Over the past 50 years, the absolute and relative (% energy) availability of sugar per capita has remained relatively stable while total food energy available for consumption has steadily increased [19,20].

Unlike other nutrients, there is no quantitative recommendation for total or added sugars intake in Canada. The Institute of Medicine, Dietary Reference Intakes (DRIs) report (2005) which forms the basis of Canada-US dietary guidance concluded that there was insufficient evidence to set an upper limit for total or added sugars based on the available data on dental caries, behavior, cancer, risk of obesity, and risk of hyperlipidemia [10]. Instead, a maximum intake of 25% of energy from added sugars was suggested based on the decreased intake of some micronutrients of American subpopulations exceeding this level [10]. The average intake of added sugars among Canadians was estimated at about half this level in this report and similar to that recommended by the World Health Organization for a population average (10%) [21]. The estimated range of intakes among different population subgroups was below the DRI 25% suggested maximum.

This study presents a best estimate of added sugars consumption but has several limitations, mainly due to the gaps in Canadian food composition data and the assumptions required to estimate added sugars intakes. No analytical methods currently exist to distinguish between added and naturally occurring sugars in foods as they are chemically identical and metabolized the same way. The most comprehensive analysis of naturally occurring and added sugars intakes undertaken in North America was that by US Food and Drug Administration, Sugars Task Force which estimated added sugars based on total sugars intake from dietary surveys. Added sugars accounted for approximately 50% of total sugars intake [22], similar to our estimates. The USDA more recently attempted to develop a database for estimates of added sugars content of select foods [23]; this database has since been withdrawn due to constant changes in formulations for commercial, multi-ingredient foods [24]. Nonetheless, their estimates for added sugars for foods were similar to the estimates derived from our division of food categories based on the majority of sugars being either added or naturally occurring, resulting in the contribution from added and naturally occurring sugars to be more or less equal in the diet, especially among adults. The adjustments for children and adolescents based on the division of the top sources were in agreement with previous studies, which report children receive a greater percentage of sugars from naturally occurring sources and adolescents from added sugars [13,16,18]. It was a limitation for our data to be restricted to the top 10 sources of sugars in the Canadian diet. However, foods not in the top 10 sources would include a variety of foods containing both naturally occurring and added sugars (e.g., yogurts, ice milk/ice cream, malted milk, instant breakfast, meal replacements, cheese and other dairy products, breads, cookies and other baked products, nuts, seeds and legumes, jams, soups, sauces, salad dressings and alcoholic beverages), which individually would each contribute less than 3% to total sugars intake and less than 1% to total energy (to collectively account for the remaining 15% total sugars). Since the contribution of each of these remaining categories would be so small, it is unlikely that the ratio of added sugars compared to naturally occurring sugars would be significantly changed from the ratio obtained from the division of the top 10 sources.

Our estimates of added sugars intake from availability data and intake data are remarkably close. However the availability data have potential for large error as they are based on two assumptions. First, the largest error may have arisen from the estimates of wastage. Food waste has been estimated to have increased by 50% since the 1970s [2]. Slightly higher estimates from availability data reported in this study may be in part due to the 40% waste adjustment factor used, which may still be too low [2]. On the other hand if we had used previous estimates of wastage of 29%, the estimated intakes for “*sugars and syrups*” would have been 60 g/day (instead of 51 g/day). Interpretation of the results when expressed as a percentage of total energy (*i.e.*, 13%) is not dependent on the wastage factor; however, this assumes the waste for all foods and beverages is the same, which may not be the case.

Second, because Canadian economic data for “*sugars and syrups*” availability does not include corn sweeteners, the use of soft drink availability data as a proxy for corn sweetener trends was an assumption and may be an underestimate. There is much lower use of HFCS and corn sweeteners in Canada compared to the US because of lower soft drink consumption as well as the difference in sugar policies between the two countries—the US support-price for sugar generally provides a greater economic incentive to use HFCS and corn sweeteners in certain food applications. For example, liquid sugar which competes with corn sweeteners in Canada represents 22% of total sugar use compared to 12% in the US [25,26]. In Canada, HFCS is predominantly used in soft drinks and there is no means to estimate use of HFCS in other foods; however, usage of corn sweeteners other than HFCS is 12% in the US. Further, Canadian soft drink availability data overestimates HFCS use (and caloric availability from soft drinks) because the data includes both regular and diet soft drinks [8], but we could not adjust for this as the distribution is unknown in Canada. US availability data show the population share of soft drinks from diet drinks to be 29% in 2004 and 31% in 2007 (when data was discontinued) [9]. From CCHS data, diet soft drinks contributed approximately 30% to total soft drink consumption among Canadian adults in 2004 [27] and ranged from 4% to 20% for adolescents [28], but this estimate may be low given the increase in diet soft drink consumption in recent years [29]. The overestimation of HFCS in soft drinks due to diet drinks however may balance the use of corn sweeteners in other foods not captured in this analysis. The use of soft drink data as a proxy for corn sweetener consumption appears reasonable, as intake data from the CCHS was similar to the consumption estimates derived from availability data. In fact, estimates from availability data were slightly higher. CCHS data reported soft drink consumption by Canadians of all ages to be 2.2% of daily calories [27,28], while availability data for the same time period (2004) indicated that soft drinks accounted for 3.5% of total energy [8]. Similarly, added sugars were estimated to account for 11% of total energy for all Canadians using food intake data compared to 13% of total energy using availability data.

## 5. Conclusions

Analysis of published data on added sugars availability and estimated consumption from the CCHS nutrition survey indicated that added sugars contribute approximately 11%–13% of total daily calories among Canadians. Intakes of added sugars among different population subgroups were estimated to range from 10% to 14% of energy intake, with higher intakes among adolescents. Availability data suggest added sugars intakes to be stable or modestly declining as a percent of total energy over the past three decades. Although these data have to be considered best estimates based on available data, this analysis may encourage the development of better databases and analyses in the future. A better understanding of added sugars consumption and trends is an essential input to evidence based public policy recommendations.
